# Parent Child Interaction Training (PCIT) in Zurich, Switzerland - Experiences and Results of the first two years

**DOI:** 10.1192/j.eurpsy.2022.1087

**Published:** 2022-09-01

**Authors:** M. Zulauf-Logoz, V. Mailaender Zelger

**Affiliations:** 1 University of Zurich Psychiatric Hospital, Child And Adolescent Psychiatry, Zurich, Switzerland; 2 University of Zurich Psychiatric Hospitly, Child And Adolescent Psychiatry, Zurich, Switzerland

**Keywords:** oppositional-defiant disorder, parent-child-interaction-training, Children, preschoolers

## Abstract

**Introduction:**

Parent Child Interaction Training PCIT (Zisser & Eyberg, 2010; Briegel, 2016) is an evidence-based treatment of oppositional defiant disorder in preschool children. However, it is implemented in few institutions in Europe. The advantage of PCIT is the involvement of both child and parents with direct coaching of the parents.

**Objectives:**

We will give an overview of 20 treatment courses we have conducted since the introduction of PCIT at the Zurich University Hospital for Child and Adolescent Psychiatry. First, a descriptive analysis of our sample will be conducted. The influence of sample characteristics and intensity of training on the reduction of behavioral problems will be analyzed.

**Methods:**

Parents attend the training for at least 6 months with regular sessions. The transfer into everyday life is achieved by daily homework. We use the Eyberg Child Problem Behavior Inventory as pre-post measurements. The ECBI is filled out by the parents before each session and also enables a progress analysis.

**Results:**

Three times more boys participated than girls. Problem behavior was significantly reduced after the play training phase. There was also a significant overall pre-post effect. The effect seemed to be independent of the parental problem score before training and of the number of play-training sessions.

**Conclusions:**

Parents and children clearly benefit from the play training. For the following cooperation training, the problem load experienced by the parents seems to be more relevant than the intensity of the child’s problem behavior as assessed by them. This is to be examined in the future.

**Disclosure:**

No significant relationships.

